# Circulating Tenascin-C/-miR-155-5p Identified as Promising Prognostic Candidates of Intervertebral Disc Herniation

**DOI:** 10.3390/bioengineering13010074

**Published:** 2026-01-08

**Authors:** Catarina Correia, Cláudia Ribeiro-Machado, Joana Caldeira, Inês C. Ferreira, Hugo Osório, Mário A. Barbosa, Milton Severo, Carla Cunha

**Affiliations:** 1 i3S—Instituto de Investigação e Inovação em Saúde, Universidade do Porto, 4200-135 Porto, Portugal; catarinapenacorreia@gmail.com (C.C.);; 2 INEB—Instituto Nacional de Engenharia Biomédica, Universidade do Porto, 4200-135 Porto, Portugal; 3 IPATIMUP—Instituto de Patologia e Imunologia Molecular, Universidade do Porto, 4200-135 Porto, Portugal; 4 Department of Pathology, Faculty of Medicine, University of Porto, 4200-319 Porto, Portugal; 5 ICBAS—Instituto de Ciências Biomédicas Abel Salazar, Universidade do Porto, Rua Jorge de Viterbo Ferreira 228, 4050-313 Porto, Portugal; 6 EPIUnit—Instituto de Saúde Pública, Universidade do Porto, Rua das Taipas 135, 4050-600 Porto, Portugal; 7 Laboratory for Integrative and Translational Research in Population Health (ITR), Rua das Taipas 135, 4050-600 Porto, Portugal

**Keywords:** circulating biomarkers, intervertebral disc, disc herniation, proteomics, miRNA

## Abstract

Intervertebral disc (IVD) herniation is a complex and multifactorial condition with a challenging diagnosis and limited therapeutic options, highlighting the need for reliable biomarkers to improve clinical decision-making. The aim of this study was to identify circulating prognostic biomarkers of IVD herniation regression. The plasma proteomic profile and the expression of circulating non-coding RNAs were analysed in a rat model of IVD herniation and were correlated with herniation size. Four candidate proteins (TNC, COPS3, JUP, and GNAI2) were significantly correlated with herniation size, with TNC further validated by ELISA. Additionally, miR-143-3p, miR-10b-5p, miR-27a-3p, miR-140-5p, miR-155-5p, miR-146a-5p, and miR-21-5p were positively correlated with herniation size. Moreover, TNC, COPS3, JUP, and GNAI2 were found to be potential targets of miR-155-5p. This study provides the first combined proteomic and miRNA account of preclinical plasma biomarkers of IVD herniation size, where TNC-miR-155-5p emerge as promising elements of a regulatory module with IVD herniation prognostic potential.

## 1. Introduction

Intervertebral disc (IVD) degeneration and subsequent herniation are the predominant causes of low back pain, primarily due to increased inflammatory responses and the stimulation of nerve fibres. This condition is the leading cause of disability worldwide and the most common reason for spinal surgery. IVD herniation (IVDH) has a peak incidence between the third and fifth decades, thus resulting in a significant socioeconomic burden, particularly due to work absenteeism [1].

Spontaneous regression of IVDH following conservative treatment is a clinically relevant phenomenon [2], but there are presently no biomarkers to predict herniation regression in clinical practice. Currently, IVDH is diagnosed and treated based on the integration of history, physical examination, and radiological evidence from magnetic resonance imaging (MRI) [3]. In particular, it is accepted that larger hernias may benefit more from conservative strategies in cases where there is no significant neurological compromise [4]. The immune system has a central role in IVD herniation regression by initiating a cascade of inflammation, angiogenesis, and extracellular matrix (ECM) remodelling that leads to tissue resorption [5].

However, due to its complexity and multiple clinical presentations, there is currently no consensus regarding disease prognosis or optimal treatment strategy—although surgical intervention is well supported for herniations with pronounced neurological deficits and favourable outcomes—while international guidelines provide poor or insufficient evidence [6], resulting in suboptimal treatment outcomes [7], particularly in cases with predominant low back pain or minor neurological impairments.

Currently, no biomarker exists to guide the selection of the best treatment option for IVD herniation management. Circulating molecular biomarkers found in plasma hold great potential for patient stratification and aiding clinical decision-making. Specifically, prognostic biomarkers that can predict the disease course offer a substantial opportunity to be explored as the foundation of IVDH personalised therapies. Building on this, systemic molecules with altered expression during the IVD degenerative process have already been identified, highlighting the potential for these biomarkers to inform and refine therapeutic approaches [8].

Mass spectrometry (MS)-based proteomics has become a powerful technology in biological research, due to its functional relevance, allowing the characterisation of the plasma proteome in great depth. Proteomic biomarkers provide a broad spectrum of protein changes, reflecting real-time physiological states and enabling early disease detection and monitoring of the treatment response [9]. Likewise, microRNAs (miRNAs) have huge potential as biomarkers for disease diagnosis and prognosis in several diseases due to their stability in circulation and their role in cell–cell communication through extracellular fluids. These are small non-coding RNAs that regulate gene expression by binding to a target mRNA, leading to translation inhibition or mRNA degradation [10]. On top of that, they could have a direct impact on modulating target protein expression and participating in regulatory modules with key roles in disease progression, as previously shown for liver fibrosis [11] and other human diseases [12]. Given the complexity of IVD herniation, an integrative omics approach will likely be necessary to identify reliable biomarkers.

This study aimed to identify plasma proteins and circulating miRNAs associated with herniation size in order to propose minimally invasive biomarkers of IVD herniation size and putative predictors of herniation regression. A biobank of herniated tissues and respective plasma samples, representing a broad range of conditions, were therefore analysed. These were collected from a previous study devoted to the development of a macrophage-based therapy for IVD herniation regression [13] in which a rat model of IVD herniation was used to test complementary experimental setups: (1) macrophage systemic depletion via intravenous administration of clodronate liposomes and (2) administration of bone marrow-derived macrophages into the herniated IVD. This previous study demonstrated the feasibility of a macrophage-based immunotherapy for IVD herniation and provided IVD tissues with a range of herniation sizes (treatment with clodronate liposomes, demonstrated to increase herniation size; treatment with macrophage administration, demonstrated to reduce herniation size) and also the respective plasma from the same animals.

## 2. Materials and Methods

**Figure 1:**
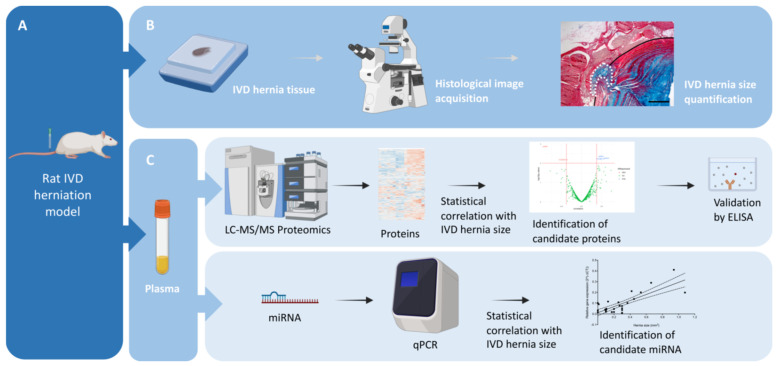
Overview of the study design. (**A**) Male Lewis (LEW/Crl) rats were subjected to IVD lesion for the induction of IVD herniation and sacrificed after 2 and 6 weeks post-injury. (**B**) The herniated tissue was collected and processed for paraffin embedding and the herniation sizes were quantified. Scale bar: 500 µm. (**C**) From the same animals, plasma was collected for proteomic and miRNA analysis. Created in Biorender.

In view of minimally invasive biomarkers of IVD herniation size and predictors for herniation regression, a complementary three-step approach was followed (Figure 1). First, an animal IVD herniation model (A) that allowed the collection and quantification of herniated tissue (B) was established. Next, plasma obtained from whole blood was analysed and proteins and miRNAs identified. Later their association to IVD herniation size was assessed by volcano plot analysis for partial correlation and regression modelling based on random forest and Pearson correlations, respectively (C). Each step is detailed hereafter.

### 2.1. Animal Model of IVD Herniation

Male Lewis (LEW/Crl) rats at 2–3 months of age were used for the IVD caudal herniation model, as previously described [5]. This inbred strain was used to reduce possible allogeneic cell rejection. Briefly, the animals were anaesthetized by isoflurane inhalation, placed in a prone position, and their tail skin was disinfected with ethanol. A percutaneous 21-G needle puncture at 5 mm depth was performed in the coccygeal IVDs Co5/6, Co6/7, and Co7/8, using radiography for IVD identification. Experiments were carried out at i3S—Instituto de Investigação e Inovação em Saúde animal facility, approved by the local Animal Ethics Committee and by Direcção Geral de Alimentação e Veterinária through licence nº 122395/24-S 22-10-2024, and conducted in accordance with the European Legislation on Animal Experimentation through Directive 2010/63/UE. The plasma samples used in this study were collected from a previous experiment devoted to the development of a macrophage-based therapy for IVD herniation regression [13]. One group of animals received macrophage administration at 2 weeks post-lesion and was sacrificed at 6 weeks post-lesion (Group Mac6w, *n* = 6). Another group of animals received clodronate liposomes between lesion and 2 weeks post-lesion (Group CLP2w, *n* = 6) or between 2 and 6 weeks post-lesion (CLP6w, *n* = 5). Another group of animals received only lesion with no treatment and was sacrificed at either 2 weeks (Group L2w, *n* = 9) or 6 weeks (Group L6w, *n* = 9) post-lesion. One Naïve rat was also analysed within the functional bioinformatic analysis.

### 2.2. IVD Histology and Quantification of Herniation Area

Each functional spinal unit (one IVD and two adjacent vertebrae) was collected en bloc and fixated in 10% neutral buffered formalin for 1 week at room temperature. The tissue was decalcified in EDTA-glycerol solution and processed for paraffin embedding. Sequential transversal 5 µm sections of the IVD were collected. Sections were stained with Alcian blue/Picrosirius red (AB/PSR) as follows: sections were deparaffinized, rehydrated, incubated in Weigert’s Iron Hematoxylin for 10 min, washed in tap water, and stained in Alcian blue solution pH 2.5 for 30 min. After rinsing in tap water, sections were incubated in Picrosirius red solution (0.1 g Sirius red in 100 mL of saturated aqueous picric acid solution) for 1 h, followed by a wash in 0.01N HCl for 2 min. Sections were dehydrated, mounted with Entellan (Merck KGaA, Darmstadt, Germany), and analysed in a CX31 optical microscope equipped with a DP25 digital colour camera (Olympus, Tokyo, Japan). The herniated area was determined as described before [5] by delimitating regions of interest (ROI) in each optical section, using the freehand selection tool in the ImageJ software (v2.0.0-rc-69/1.52p), considering blue staining for proteoglycans and red staining for collagen. The respective percentage of area was determined by a custom ImageJ macro based on a colour deconvolution technique used to separate the different colour channels [14]. The mean herniated area (mm^2^) for each animal was calculated as the mean areas of each individual section throughout the IVD (Equation (1), where *A_ij_* = the *j*^th^ measured area for section *i*; *m* = total number of measurements).

(1) $$Mean\;herniated\;area\;\left({\mathrm{mm}}^{2}\right)=\frac{1}{m}\sum_{j=1}^{m}A_{ij}$$

### 2.3. Collection of Plasma from Whole Blood for Proteomic and miRNAs Analysis

Total blood was collected by intracardiac puncture, under isoflurane anaesthesia, into anti-coagulant EDTA solution. Blood was centrifuged at 800 *g* for 20 min at room temperature and plasma was collected. Plasma was further centrifuged twice at 2500 *g* for 15 min at 4 °C to remove cell debris and kept at −80 °C for proteomic and miRNAs analysis.

### 2.4. Plasma LC-MS/MS Analysis for Protein Identification

Plasma was centrifuged at 15,000 *g* for 15 min at 4 °C, total protein was quantified by the BCA method, and 100 µg of total protein was solubilized and processed for proteomic analysis following the solid-phase-enhanced sample preparation (SP3) protocol as described [15]. After enzymatic digestion, protein identification and quantitation were performed by label-free high-resolution accurate-mass nanoLC-MS/MS, which is composed of an Ultimate 3000 liquid chromatography system coupled to a Q-Exactive Hybrid Quadrupole-Orbitrap mass spectrometer (Thermo Fisher Scientific, Waltham, MA, USA), as described in [16]. Data acquisition was controlled by Xcalibur 4.0 and Tune 2.11 software (Thermo Fisher Scientific, Waltham, MA, USA).

### 2.5. Database Search

LC-MS/MS raw data was processed using Proteome Discoverer 2.5.0.400 software (Thermo Fisher Scientific, Waltham, MA, USA) and searched against the UniProt database for the *Rattus norvegicus* Proteome 2020_05 and a predicted spectral library. A common protein contaminant list from MaxQuant was also considered. The Sequest HT search engine was used to identify tryptic peptides. Data was filtered to consider only proteins with unique peptides ≥ 2. However, proteins with unique peptides = 1 and razor peptides ≥ 2 were also included. Furthermore, proteins with unique peptides = 1 and razor peptides = 1 were kept if they had a low molecular weight (<10 kDa). All false discovery rates (FDRs) were considered.

### 2.6. Volcano Plot Analysis

Volcano plot analysis for partial correlation (adjusted for time and treatment) between protein abundance and herniation size was performed, with –log10 (*p*-value) on the *y*-axis and magnitude of correlation on the *x*-axis. A significance level of 0.01 was used, and effect magnitude was considered as moderate partial correlation when <−0.3 or >0.3.

### 2.7. Random Forest Regression Model

A robust two-step modelling approach was used to predict the association between plasma protein abundance and herniation size. First, a linear regression model was performed with treatment and time as predictors of herniation size, and the residuals of the model, or the proportion of the herniation size that was not explained by treatment and time, were saved as a new variable. Second, a random forest regression model with 3-fold cross-validation with 50 repetitions evaluated the contribution of plasma protein abundance to herniation size, using the residuals of the linear regression as the dependent variable and protein abundance as the explanatory variable. By predicting the values of the residuals, the random forest model identified associations between plasma protein abundance and the portion of the herniation size that is independent of the effects of treatment and time. The random forest model produced a variable importance ranking based on the increase in node purity (IncNodePurity) that was used to verify whether the previously identified protein abundances were also selected as the best predictors for herniation size. Proteins with higher IncNodePurity values are those that are most influential in predicting the variance of the residuals, which indicates a strong association with herniation size after accounting for treatment and time. IncNodePurity represents the total increase in node purity from splitting on each variable, averaged across all trees (using the Gini coefficient as a measure). In regression, node purity is measured by the residual sum of squares. We also included the ranking of variables from the worst to the best based on IncNodePurity. Random forest regression is a nonparametric statistical ensemble method that makes no distributional, functional (linear or non-linear), or interaction effect assumptions about covariate relationships to predict the response. The R Studio package “RandomForest” (v4.4.0) [17] was used to develop the random forest model.

### 2.8. Enzyme-Linked Immunosorbent Assays (ELISA) Analysis

Each of the following enzyme-linked immunosorbent assays (ELISA) was acquired from MyBioSource (San Diego, CA, USA) and performed according to the suggested protocol in the *n* = 35 samples under study. Rat Tenascin C (TNC) ELISA Kit (Sensitivity: 18.75 pg/mL); Rat Guanine nucleotide-binding protein G (GNAI2) ELISA Kit (Sensitivity: 31.25 pg/mL); Rat Junction plakoglobin (JUP) ELISA Kit (Sensitivity: 78.0 pg/mL); Rat COP9 signalosome complex subunit 3 (COPS3) ELISA Kit (Sensitivity: 0.1 ng/mL).

### 2.9. Functional Bioinformatic Analysis

For the identification of differentially abundant proteins (DAPs), only the Lesion Groups (Group L2w, Group L6w, *n* = 18) and Naïve (*n* = 1) were analysed. Proteins with an abundance ratio (Lesion/Control) < 0.5 were considered to be down-regulated, and proteins with an abundance ratio (Lesion/Control) > 2.0 were considered to be up-regulated. Morpheus (https://software.broadinstitute.org/morpheus/, accessed on 29 September 2025) was used for data analysis and visualisation, and STRING database v12.0 (https://string-db.org/) was used for the construction of PPI networks.

### 2.10. Isolation and Quantification of Circulating microRNAs (miRNAs)

miRNAs from plasma were isolated with the miRNeasy Serum/Plasma Kit (Qiagen N.V., Hilden, Germany) according to the manufacturer’s instructions. Briefly, 0.4 mL of TRIZol Reagent was added to 200 µL of plasma. After strong agitation, two exogenous C. elegans miRNAs spike-in controls (cel-miR-39-3p and cel-miR-54-3p) were added to the reaction to serve as internal standards for accurate quantification and quality control. Then, 200 µL of chloroform was added and the samples were centrifuged at 12,000 *g* for 15 min at 4 °C. The upper aqueous phase was collected and mixed with ice-cold 100% ethanol. Samples were transferred to the RNeasy MinElute spin columns and washed, and the RNA was eluted in 30 µL of RNase-free water. Finally, the RNA concentration was determined using the Bioanalyzer Small RNA Analysis (Agilent Technologies, Inc., Santa Clara, CA, USA). RNA was converted to cDNA through the TaqMan^®^ Advanced miRNA cDNA Synthesis Kit (Applied Biosystems, Foster City, CA, USA), according to the manufacturer’s protocol, including the poly(A) tailing reaction, the adaptor ligation reaction, the reverse transcription reaction, and the miR-Amp reaction. Then 2.5 µL of cDNA were mixed with TaqMan Fast Advanced Master Mix (Applied Biosystems, Foster City, CA, USA); the TaqMan Advanced miRNA Assays for rno-miR-21-5p, rno-miR-10b-5p, rno-miR-27a-3p, rno-miR-93-5p, rno-miR-100-5p, rno-miR-127-5p, rno-miR-132-3p, rno-miR-140-5p, rno-miR-143-3p, rno-miR-146a-5p, rno-miR-155-5p and rno-miR-377-3p; and nuclease-free water in a 7500 Fast Real-Time PCR System (Applied Biosystems, Foster City, CA, USA) and incubated as follows: 95 °C for 20 s; 40 cycles of 95 °C for 3 s and 60 °C for 30 s. qPCR reactions were performed in duplicates. The results were analysed in the 7500 Software v2.0.6 (Applied Biosystems, Foster City, CA, USA). Exogenous cel-miR-39-3p and cel-miR-54-3p were used as normalizers. The relative gene expression was calculated using the ΔCq method, in accordance with MIQE guidelines [18].

### 2.11. Statistical Data Analysis

Data normality was assessed by the D’Agostino-Pearson omnibus normality test. Pearson’s bivariate correlation was performed to link the expression of each specific miRNA in the plasma to herniation size. Statistical analysis was performed with the nonparametric Kruskal–Wallis test, followed by Dunn’s multiple comparison test, using GraphPad Prism 7 (v7.0a). Statistical significance was set at *p* < 0.05.

## 3. Results

**Figure 2:**
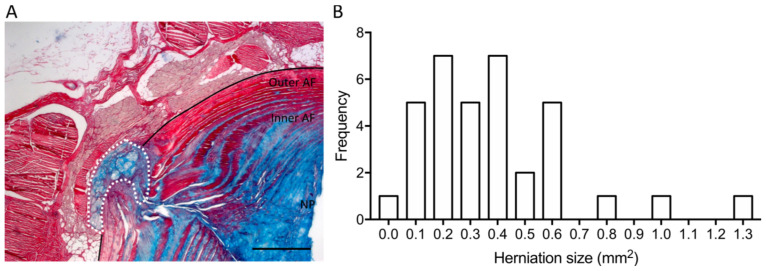
(**A**) Representative IVD section, stained blue for proteoglycans (Alcian blue) and red for collagen (Sirius red), showing the herniation (white dashed line) within the structure of the IVD: annulus fibrous (AF) and nucleus pulposus (NP). Scale bar: 500 µm. (**B**) Frequency distribution of rat herniation sizes (*n* = 35).

In order to identify putative biomarkers associated with IVDH, mass spectrometry (MS)-based proteomic and miRNA analyses were performed in *n* = 35 rat plasma samples collected from different experimental setups (Figure 1). Specifically, samples were obtained by performing a lesion in the rats’ caudal IVDs [5] and applying different therapies at different time points as follows: one group of animals received macrophage administration (demonstrated to reduce herniation size) at 2 weeks post-lesion and was sacrificed at 6 weeks post-lesion (Group Mac6w, *n* = 6). Another group of animals received clodronate liposomes (demonstrated to increase herniation size) between lesion and 2 weeks post-lesion (Group CLP2w, *n* = 6) or between 2 and 6 weeks post-lesion (CLP6w, *n* = 5). Another group of animals received only a lesion with no treatment and was sacrificed at either 2 weeks (Group L2w, *n* = 9) or 6 weeks (Group L6w, *n* = 9) post-lesion [13]. From these groups, a distribution of herniation sizes was obtained, ranging from 0.0135 to 1.2932 mm^2^ (Figure 2).

### 3.1. Identification of Candidate Proteins Correlated with Herniation Size

The MS-based proteomic analysis identified a total of 1055 proteins by searching against the UniProt database for the *Rattus norvegicus* Proteome 2020_05 and a predicted spectral library. After removal of contaminants and data filtering, 674 proteins were selected for analysis.

**Figure 3:**
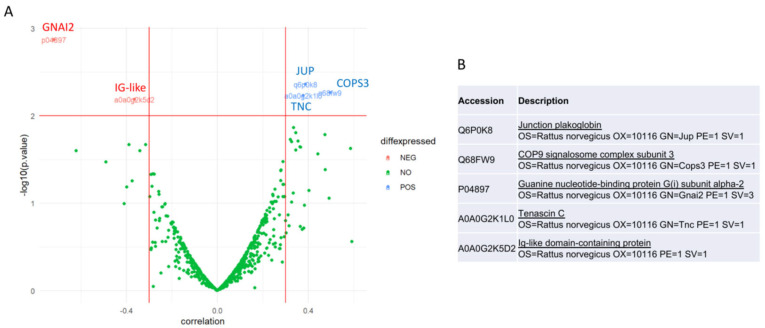
Identification of candidate plasma proteins correlated with herniation size in *n* = 35 plasma samples. (**A**) Volcano plot for partial correlation (adjusted for time and treatment) between protein abundance and herniation size, showing the distribution and significance of each bivariate association. The *y*-axis represents the logarithm (base-10) of the *p*-value of each correlation coefficient. The *x*-axis represents the correlation coefficient. The horizontal red line is the Bonferroni-adjusted *p*-value, considered as the significance level of 0.01. Effect magnitude is considered as moderate partial correlation when <−0.3 or >0.3. Selected proteins are labelled with accession numbers on the plot. (**B**) Accession number and respective protein name for the 5 proteins identified by the volcano plot.

To link plasma protein abundances to herniation size, a volcano plot analysis based on partial correlation was performed, followed by regression modelling based on a random forest. The volcano plot analysis highlighted the proteins whose abundance had the strongest and most significant association with herniation size, after controlling for the effects of time and treatment, in order to exclude possible confounding factors given by these variables. A moderate partial correlation was considered positive when <−0.3 or >0.3 [19] (Figure 3). Five proteins were identified as correlating with herniation size: Guanine nucleotide-binding protein G(i) subunit alpha-2 (GNAI2) and Ig-like domain-containing protein were negatively correlated with herniation size, and Junction plakoglobin (JUP), COP9 signalosome complex subunit 3 (COPS3), and Tenascin C (TNC) were positively correlated with herniation size.

**Figure 4:**
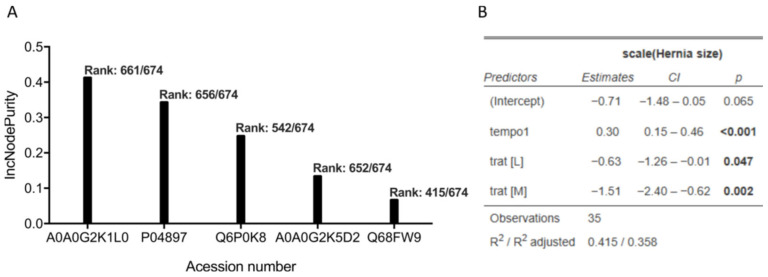
(**A**) Variable importance (IncNodePurity) estimated by the random forest prediction model, demonstrating the contributions of TNC (A0A0G2K1L0), GNAI2 (P04897), JUP (Q6P0K8), Ig-like domain-containing protein (A0A0G2K5D2), and COPS3 (Q68FW9) on the prediction of herniation size (mm^2^), independent of the effects of treatment and time. The random forest analysis used the residuals of the linear regression (the unexplained herniation size) with treatment and time as predictors of herniation size (outcome). Proteins with higher IncNodePurity values are those that are most influential in predicting the variance of the residuals, which indicates a strong association with herniation size after accounting for treatment and time. (**B**) Linear regression modelling was performed to remove the effect of treatment and time. All analyses were performed by the random forest package (R Studio version 4.4.0). *n* = 35.

To assess the predictive power and importance of the identified candidate proteins for predicting herniation size, a random forest regression analysis was conducted. The residuals were extracted from a linear regression analysis that adjusted the herniation size for time and treatment. This new variable was then used as an outcome on a random forest where all the proteins were considered exposures. From the random forest, we calculated the importance plot that showed the most important variables for the prediction of the outcome. Then, the five most important proteins were selected (Figure 4).

**Figure 5:**
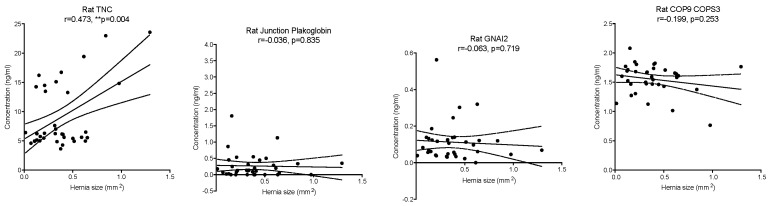
Validation of protein candidates by ELISA for Rat Tenascin C (TNC), Rat Guanine nucleotide-binding protein G (GNAI2), Rat Junction plakoglobin, and Rat COP9 signalosome complex subunit 3 (COPS3). ** *p* ≤ 0.01, *n* = 35.

The results indicate that the previously identified proteins indeed have a high predictive power, indicating a node purity of 661 out of 674 for TNC (A0A0G2K1L0), 656 out of 674 for GNAI2 (P04897), 542 out of 674 for JUP (Q6P0K8), 652 out of 674 for Ig-like domain-containing protein (A0A0G2K5D2), and 415 out of 674 for COPS3 (Q68FW9). Apart from Ig-like domain-containing protein, which is a generic predicted protein, the remaining four candidate proteins were analysed by ELISAs, with TNC being validated (r = 0.473, *p* = 0.004), while the remaining were not validated: JUP (r = −0.036, *p* = 0.835), GNAI2 (r = −0.063, *p* = 0.719), COPS3 (r = −0.199, *p* = 0.253) (Figure 5). 

**Figure 6:**
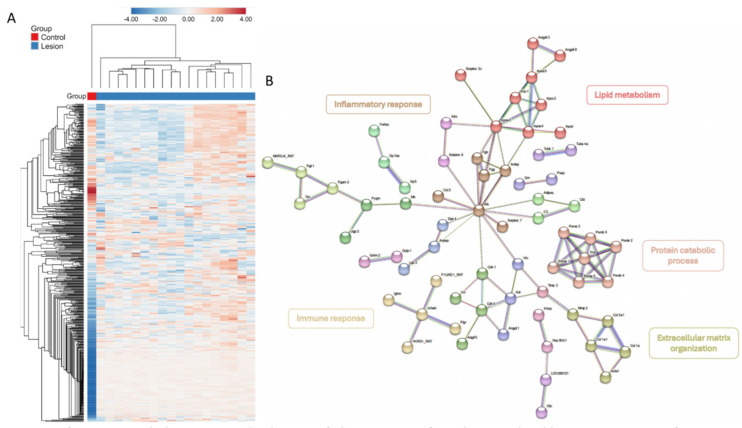
(**A**) Heatmap with the respective dendrogram of plasma proteins from the Control (*n* = 1) and Lesion groups (*n* = 18). A transformation to ln (x + 1) and a scaling of the unit variance were used. Hierarchical clustering was performed using the linkage method average with one minus Pearson correlation as a metric for the rows and the linkage method average with Euclidean distance as a metric for the columns. (**B**) Protein–protein interaction (PPI) networks of differentially abundant proteins (DAPs) down- and up-regulated between the Lesion and the Control groups. K-means clustering was performed. The minimum required interaction score was set to 0.700 (high confidence), and disconnected nodes were hidden in the network. The nodes represent the proteins and the edges represent the interactions between them. Functionally related clusters were identified and highlighted with different colours.

Functional bioinformatic analysis was conducted for the identification of key pathways involved in IVD herniation by comparing the Lesion and Naïve groups. A heatmap with hierarchical clustering indicated distinct proteomes between Lesion and Naïve groups (Figure 6A). Differentially abundant proteins (DAPs) were identified between Lesion and Naïve groups, and a protein–protein interaction (PPI) network was constructed using STRING database (v12.0). The obtained PPI network reveals that the interactome is composed of 19 distinct clusters, each with distinct functions (Figure 6B). For the clusters with the largest number of proteins, the main functions were graphically highlighted and are predominantly associated with the biological processes of inflammatory response, immune response, lipid metabolism, protein catabolic process, and ECM organisation.

### 3.2. Identification of Candidate miRNAs Correlated with Herniation Size

**Figure 7:**
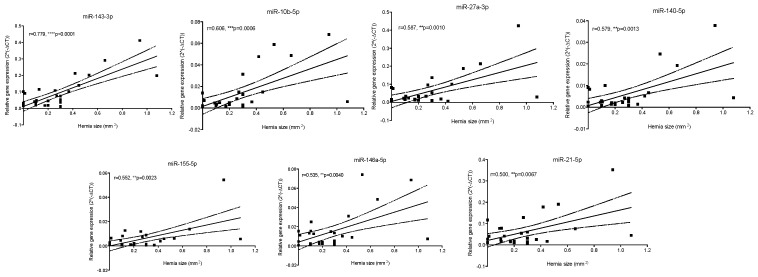
Correlation between herniation size (mm^2^) and plasma miRNAs expression levels. For each miRNA, the Pearson correlation coefficient and significance level (*p*-value) are shown. ** *p* ≤ 0.01, *** *p* ≤ 0.001, **** *p* ≤ 0.0001, *n* = 35.

A panel of candidate miRNAs was selected from the literature based on previous reports of their presence in IVD tissue: miR-21-5p [20], miR-27a-3p [21], miR-93-5p [22], miR-100-5p [23], miR-127-5p [24], miR-132-3p [25], miR-140-5p [26], miR-143-3p [27], miR-146a-5p [28], miR-155-5p [29], and miR-377-3p [30]. Moreover, miR-10b-5p was also analysed to investigate a possible association with IVD herniation. Pearson’s correlation was performed between herniation size and the expression of each specific circulating miRNA in the plasma. The results indicate that miR-143-3p (r = 0.779), miR-10b-5p (r = 0.606), miR-27a-3p (r = 0.587), miR-140-5p (r = 0.579), miR-155-5p (r = 0.552), miR-146a-5p (r = 0.535), and miR-21-5p (r = 0.500) have a significant positive correlation with herniation size (Figure 7). Alternatively, miR-93-5p, miR-100-5p, miR-127-5p, miR-132-3p, and miR-377-3p did not show any significant correlation with herniation size.

## 4. Discussion

This study found four circulating proteins potentially associated with herniation: Guanine nucleotide-binding protein G (GNAI2), Junction plakoglobin (JUP), COP9 signalosome complex subunit 3 (COPS3), and Tenascin C (TNC); however, only TNC was validated by ELISA. TNC is a highly conserved ECM glycoprotein found in many stem cell niches and it is known to contribute to several functions, such as cell adhesion, tissue remodelling, and tumour cell migration and invasion, owing to the presence of alternatively spliced isoforms and the various domains present [31]. In adults, its expression is physiologically low, and its expression has been shown to be up-regulated under pathological conditions caused by inflammation, infection, and tumorigenesis and at sites that are subjected to unique biomechanical forces [32,33]. TNC is currently being researched as a potential biomarker for a number of diseases, such as myocarditis and different forms of cancer [34]. Importantly, it has recently been found to be expressed in the nucleus pulposus (NP), specifically in a progenitor NP niche [35].

Regarding the candidate miRNAs identified—miR-143-3p, miR-10b-5p, miR-27a-3p, miR-140-5p, miR-155-5p, miR-146a-5p, and miR-21-5p—these miRNAs are involved in regulating gene expression related to inflammation, ECM remodelling, and cellular apoptosis, processes that are critical in the pathogenesis of IVDH. In particular, miR-143 has been shown to promote IVD degeneration by targeting specific pathways related to inflammation and matrix degradation involving SOX5 [36] and eEF2 regulation [27]; miR-27a was shown to be involved in the regulation of apoptosis in NP cells by targeting PI3K [21] and also to be involved in exacerbating IVD degeneration by inducing M1 macrophage polarisation, which leads to increased inflammatory responses and tissue degradation [37]; miR-140 plays a protective role in cartilage and has been implicated in maintaining ECM homeostasis, which is crucial for IVD integrity [38]; decreased miR-155 has been shown to contribute to the up-regulation of MMP-16 in vivo [29] and has previously been identified as a circulating biomarker of IVD degeneration [39], but not previously linked to herniation; miR-146a is known for its anti-inflammatory properties and was shown to reduce IL-1-dependent inflammatory responses in the IVD [28] and also to be involved in IL-6/STAT3-mediated IVD degeneration [40]; miR-21-5p was shown to promote human NP cell proliferation through PTEN/AKT signalling [41].

Importantly, all four proteins identified in our study have been reported as potential targets of miRNA miR-155-5p in a transcriptome-wide identification of miR-155 targets [42]. Moreover, TNC was found to control TNF-α translation via the induction of miR-155 [43]. Also, GNAI2 was found to be a target of miR-27a-3p [44] and miR-21-5p [45]. Furthermore, COPS3 was found to be a target of miR-27a-3p and miR-146a-5p [42].

Limitations of this study include the lack of validation in human samples. A rat IVD lesion model was analysed, which does not fully replicate the multifactorial, slowly progressive, and patient-specific nature of human IVD herniation. Future studies should incorporate detailed analyses of human samples to improve clinical translatability. Also, the small number of samples analysed, in particular the use of only one naïve animal for the functional bioinformatic analysis, may be too low to establish baseline expression. Regarding the ELISA validation results, only a moderate correlation between TNC and IVD herniation size was found, so a higher number of samples would strengthen the analysis. Moreover, future studies including wide transcriptomic analysis may better explore a larger set of miRNAs rather than the ones selected in this study, which were retrieved based on an educated guess from the literature. Finally, this study identified correlations between IVD hernia size and protein and miRNAs; however, in order to identify prognostic biomarkers of IVD herniation progression, longitudinal follow-up studies should be conducted.

## 5. Conclusions

Proteins and miRNAs can be excellent candidates for the identification of biomarkers of IVD herniation regression. This study provides the first proteomic and miRNA account of preclinical plasma biomarkers of IVD herniation size, which may constitute promising predictors of IVD herniation progression and aid in the development of personalised therapies. This approach illustrates the power of multi-omics analyses and can be readily applicable to biomarker discovery and validation in human patients.

## Data Availability

The datasets generated during and/or analysed during the current study are available from the corresponding author on reasonable request. The proteomics datasets have been deposited in the ProteomeXchange Consortium via the PRIDE [46] partner repository with the dataset identifier reference: PXD071649.
